# Targeting Ferroptosis in Nasopharyngeal Carcinoma: Mechanisms, Resistance, and Precision Therapeutic Opportunities

**DOI:** 10.3390/ijms262311439

**Published:** 2025-11-26

**Authors:** Jaewang Lee, Jong-Lyel Roh

**Affiliations:** 1 Department of Otorhinolaryngology-Head and Neck Surgery, CHA Bundang Medical Center, CHA University, Seongnam 13496, Republic of Korea; 2 Logsynk, Seoul 06164, Republic of Korea; 3 Department of Biomedical Science, General Graduate School, CHA University, Pocheon 11160, Republic of Korea

**Keywords:** ferroptosis, nasopharyngeal carcinoma, therapy resistance, biomarkers, nanomedicine

## Abstract

Nasopharyngeal carcinoma (NPC) is a head and neck malignancy strongly associated with Epstein–Barr virus (EBV) infection and characterized by high radiosensitivity but frequent therapy resistance. Despite advances in radiotherapy, chemotherapy, and immunotherapy, relapse and metastasis remain major challenges, underscoring the need for novel therapeutic approaches. This review aims to provide an integrated overview of the molecular mechanisms governing ferroptosis in NPC and to clarify how these pathways contribute to therapy resistance while revealing potential therapeutic vulnerabilities. Ferroptosis, an iron-dependent form of regulated cell death driven by lipid peroxidation, has emerged as a promising target in NPC. Core regulators include the system xCT–GSH–GPX4 antioxidant axis, iron metabolism, and lipid remodeling enzymes such as ACSL4, with epigenetic modifiers (METTL3, IGF2BP2, HOXA9) and EBV-driven signaling further shaping ferroptosis responses. EBV-driven oncogenic programs substantially reshape ferroptosis sensitivity in NPC by activating the Nrf2/Keap1 antioxidant axis, stabilizing SLC7A11 and GPX4, and modulating iron and redox metabolism. These viral mechanisms suppress ferroptotic stress and contribute to both radioresistance and chemoresistance. Suppression of ferroptosis underlies both radioresistance and chemoresistance, whereas restoration of ferroptosis re-sensitizes tumors to treatment. Natural compounds including solasodine, berberine, cucurbitacin B, and celastrol-curcumin combinations, as well as pharmacologic modulators such as HO-1 inhibitors and GPX4 antagonists, have shown ferroptosis-inducing effects in preclinical models, although their translational potential remains to be clarified. Nanotechnology-based platforms (e.g., Bi_2_Se_3_ nanosheet hydrogels) further enhance efficacy and reduce toxicity by enabling controlled drug delivery. Biomarker discovery, encompassing ferroptosis-related gene signatures, epigenetic regulators, immune infiltration patterns, EBV DNA load, and on-treatment redox metabolites, provides a foundation for patient stratification. Integration of ferroptosis modulation with radiotherapy, chemotherapy, and immunotherapy represents a compelling strategy to overcome therapy resistance. In synthesizing these findings, this review highlights both the mechanistic basis and the translational promise of ferroptosis modulation as a strategy to overcome treatment resistance in NPC. Future directions include biomarker validation, optimization of drug delivery, early-phase clinical trial development, and multidisciplinary collaboration to balance ferroptosis induction in tumors while protecting normal tissues. Collectively, ferroptosis is emerging as both a vulnerability and a therapeutic opportunity for improving outcomes in NPC.

## 1. Introduction

**Figure 1:**
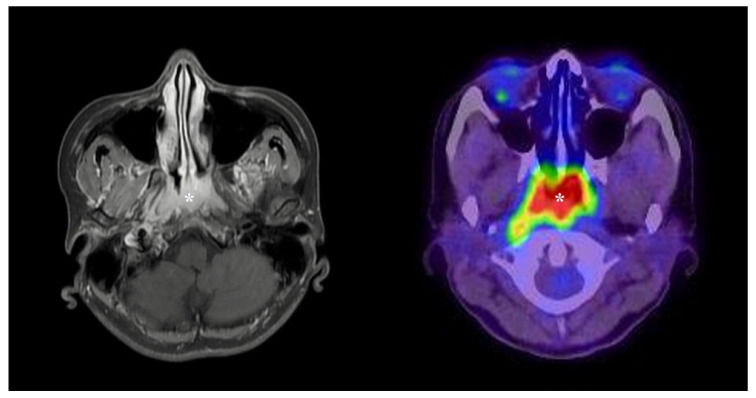
Clinical presentation and localization of nasopharyngeal carcinoma. Gadolinium-enhanced axial magnetic resonance imaging (MRI) (**left**) and ^18^F-fluorodeoxyglucose (FDG) positron emission tomography/computed tomography (PET/CT) (**right**) show a soft-tissue mass in the fossa of Rosenmüller (asterisks), with corresponding intense metabolic activity. These images illustrate the characteristic anatomical localization and imaging appearance of nasopharyngeal carcinoma (NPC).

Nasopharyngeal carcinoma (NPC) is a malignant tumor arising from the epithelial lining of the nasopharynx, typically from the fossa of Rosenmüller (Figure 1). Although NPC is relatively rare worldwide, it demonstrates striking geographic distribution, with the highest incidence in East and Southeast Asia, particularly southern China, as well as North Africa and parts of the Arctic [1]. In 2021, more than 118,000 new cases were reported globally, with over 70% occurring in endemic regions [2]. This geographic heterogeneity is explained by a multifactorial etiology. The strongest risk factor is chronic infection with Epstein–Barr virus (EBV), which integrates into the host genome and drives oncogenic programs through latent proteins, non-coding RNAs, and epigenetic reprogramming [3]. Genetic predisposition, including polymorphisms in HLA genes, further modulates susceptibility, while environmental exposures such as consumption of preserved or salted foods containing nitrosamines, occupational carcinogens, cigarette smoking, and alcohol intake contribute additional risk [4].

Clinically, NPC is distinct from other head and neck cancers. Because of its anatomical location, early-stage tumors are often asymptomatic or present with vague complaints such as nasal obstruction, serous otitis media, or epistaxis [5]. By the time of diagnosis, many patients already have cervical lymph node metastases, reflecting the tumor’s propensity for early dissemination. Radiotherapy (RT) remains the cornerstone of treatment due to the intrinsic radiosensitivity of NPC, and the advent of intensity-modulated radiotherapy (IMRT) as substantially improved local control and reduced toxicity [6]. For locoregionally advanced disease, concurrent chemoradiotherapy with cisplatin-based regimens is the standard of care [7]. In recurrent or metastatic disease, systemic chemotherapy and, more recently, immune checkpoint inhibitors targeting PD-1 have expanded therapeutic options [8]. Nevertheless, 15–20% of patients still experience local recurrence or distant metastasis despite these advances [9]. Overcoming therapeutic resistance, therefore, remains an unmet clinical need.

In parallel with these clinical challenges, ferroptosis has emerged as a unique form of regulated cell death with potential therapeutic relevance in oncology. First described in 2012, ferroptosis is mechanistically distinct from apoptosis, necroptosis, and pyroptosis, being defined by an iron-dependent accumulation of lipid peroxides within cellular membranes [10,11]. Morphologically, ferroptotic cells exhibit shrunken mitochondria with increased membrane density and loss of cristae, while the nucleus remains intact without chromatin condensation or fragmentation [12]. This phenotype reflects a biochemical imbalance: an abundance of pro-oxidant inputs and depletion or failure of antioxidant defenses.

**Figure 2:**
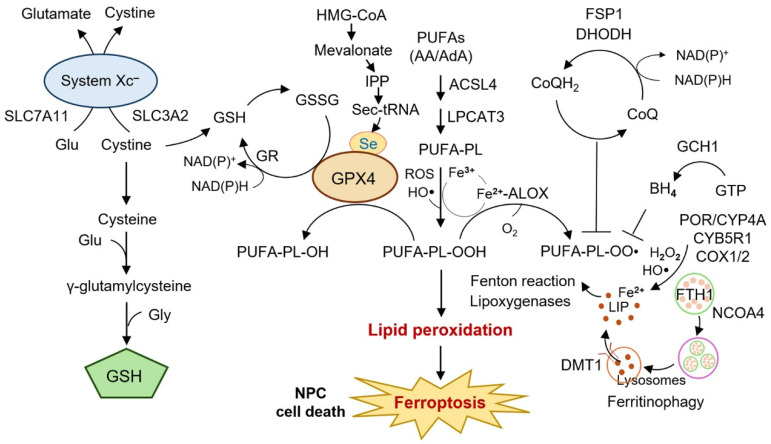
Canonical pathways and regulators of ferroptosis relevant to cancer and NPC biology. Ferroptosis is an iron-dependent form of regulated cell death characterized by excessive lipid peroxidation and distinct from apoptosis, necroptosis, and pyroptosis. The system xCT–GSH–GPX4 axis provides central antioxidant defense, while PUFAs incorporated by ACSL4 and LPCAT3 constitute vulnerable lipid substrates. Iron liberated from ferritinophagy (NCOA4–FTH1) or imported by DMT1 fuels Fenton chemistry, generating ROS that initiate lipid radical chain reactions. Enzymatic drivers such as lipoxygenases, cyclooxygenases, and POR-dependent pathways further amplify lipid peroxidation. Counteracting mechanisms include GPX4-mediated detoxification, ubiquinol regeneration via FSP1 and DHODH, and radical trapping by the GCH1–BH_4_ pathway. Failure of these protective systems results in lethal lipid peroxide accumulation and ferroptotic cell death. In nasopharyngeal carcinoma, dysregulation of these regulators, including upregulation of SLC7A11 and GPX4 or suppression of ACSL4 activity, contributes to radioresistance and chemoresistance, whereas deliberate ferroptosis induction has emerged as a promising therapeutic strategy. Abbreviations: ACSL4, acyl-CoA synthetase long-chain family member 4; ALOX, lipoxygenase; BH_4_, tetrahydrobiopterin; COX, cyclooxygenase; DHODH, dihydroorotate dehydrogenase; DMT1, divalent metal transporter 1; FSP1, ferroptosis suppressor protein-1; FTH1, ferritin heavy chain 1; GCH1, GTP cyclohydrolase 1; GPX4, glutathione peroxidase 4; GSH, glutathione; LPCAT3, lysophosphatidylcholine acyltransferase 3; NCOA4, nuclear receptor coactivator 4; NPC, nasopharyngeal carcinoma; POR, cytochrome P450 oxidoreductase; PUFA, polyunsaturated fatty acid; ROS, reactive oxygen species; Sec, selenocysteine; SLC7A11, solute carrier family 7 member 11; xCT, cystine/glutamate antiporter.

Three core elements underpin ferroptosis (Figure 2) [13]. First, reactive oxygen species (ROS) provide the initiating oxidative stress, arising from mitochondria, NADPH oxidases, enzymatic oxidoreductases such as POR, and the Fenton reaction catalyzed by ferrous iron. Second, oxidizable lipids serve as substrates, particularly polyunsaturated fatty acids (PUFAs) that are incorporated into phospholipids by acyl-CoA synthetase long-chain family member 4 (ACSL4) and lysophosphatidylcholine acyltransferase 3 (LPCAT3), rendering membranes highly vulnerable to peroxidation. Third, lipid peroxidation is driven enzymatically by lipoxygenases (ALOXs), cyclooxygenases (COXs), and cytochrome P450 oxidoreductases, as well as through non-enzymatic free radical chain reactions [13]. Together, these factors establish the lethal lipid ROS accumulation that defines ferroptotic death.

Cellular defense systems act to counteract this process [14]. Central is the system xCT–GSH–GPX4 axis: SLC7A11 imports cystine in exchange for glutamate, cystine is reduced to cysteine for glutathione (GSH) synthesis, and GPX4 reduces phospholipid hydroperoxides to non-toxic lipid alcohols, thereby preserving membrane integrity [15,16]. Beyond glutathione peroxidase 4 (GPX4), additional ferroptosis suppressors have been identified: ferroptosis suppressor protein-1 (FSP1) regenerates reduced Coenzyme Q_10_ at the plasma membrane; dihydroorotate dehydrogenase (DHODH) provides mitochondria-specific protection; and the GTP cyclohydrolase-1 (GCH1)–tetrahydrobiopterin (BH_4_) pathway contributes to lipid radical trapping [17]. Transcription factors such as NRF2, TP53, and ATF4 modulate ferroptosis sensitivity by regulating antioxidant, iron, and amino acid metabolism. Finally, membrane repair processes involving endosomal sorting complexes required for transport (ESCRT)-III complexes and lipid remodeling provide last-resort protection from ferroptotic rupture [18,19].

The significance of ferroptosis in cancer lies in its context-dependent roles. Many tumors, including NPC, evolve mechanisms to evade ferroptosis, thereby contributing to therapy resistance. For example, nuclear factor erythroid-2-related factor 2 (NRF2) activation, GPX4 overexpression, or solute carrier family 7 member 11 (SLC7A11) upregulation are common adaptive strategies. Conversely, deliberate induction of ferroptosis offers a therapeutic advantage by selectively eliminating apoptosis-resistant and therapy-tolerant cells, which are often enriched in advanced and recurrent cancers [20].

Mounting evidence highlights ferroptosis as an integral component of NPC biology. Experimental studies demonstrate that regulators such as SLC7A11, GPX4, ACSL4, heme oxygenase-1 (HO-1), and TP53 modulate radiosensitivity and chemoresistance [21,22] (Li et al., 2021; Zhou et al., 2025). Epigenetic and post-transcriptional mechanisms, including METTL3-mediated *N*6-methyladenosine (m^6^A) modification, IGF2BP2-dependent stabilization of ceruloplasmin, and HOXA9-UBR5-SIRT6 signaling, further refine ferroptotic susceptibility [23,24]. Viral and microenvironmental influences add additional layers: EBV-driven GPX4 upregulation, cancer-associated fibroblast (CAF)-derived factors, and immune cell crosstalk all act to suppress ferroptosis and promote tumor survival [25]. On the other hand, natural compounds such as berberine, cucurbitacin B, solasodine, luteolin, and celastrol-curcumin combinations have shown the ability to induce ferroptosis and inhibit NPC growth in preclinical studies [26,27,28,29]. Nanotechnology-based strategies, such as Bi_2_Se_3_ nanosheet hydrogels, are being developed to enable spatiotemporal control of ferroptosis induction, highlighting translational potential [30].

Taken together, NPC exemplifies a malignancy where conventional therapies remain limited by resistance, and ferroptosis provides both a mechanistic framework and a therapeutic opportunity. In this review, we aim to provide an integrated and comprehensive synthesis of how ferroptosis intersects with NPC biology, highlighting the molecular regulators, the impact of EBV-driven and microenvironmental programs, the mechanisms underlying radio- and chemoresistance, and emerging therapeutic and biomarker-guided strategies. By consolidating these domains, this review outlines current challenges and future directions for translating ferroptosis-based approaches into precision therapy for NPC.

## 2. Molecular Mechanisms Linking Ferroptosis to NPC

The regulation of ferroptosis in NPC involves an intricate interplay of metabolic, epigenetic, and signaling pathways that converge on lipid peroxidation, iron metabolism, and antioxidant defenses. These networks define whether tumor cells undergo ferroptotic death or evade it, thereby influencing radiosensitivity, chemoresistance, and overall tumor progression. Recent studies highlight that ferroptosis in NPC is shaped not only by canonical regulators such as the system xCT–GSH–GPX4 axis and iron metabolism but also by tumor microenvironmental signals, viral oncogenesis, and epigenetic modifications (Table 1) [13,20].

### 2.1. System x_c_^−^–GSH–GPX4 Axis

A central determinant of ferroptosis resistance in NPC is the cystine/glutamate antiporter system x_c_^−^ (xCT), which maintains intracellular cystine supply and supports GSH biosynthesis. Elevated expression of SLC7A11 is frequently observed in NPC cells, enhancing cystine uptake and GSH synthesis, thus buffering lipid peroxidation and protecting cells from ferroptosis [21]. GPX4, a selenoenzyme that reduces phospholipid hydroperoxides, acts as the executioner defense, and its upregulation in NPC correlates with poor prognosis and therapy resistance [43]. Pharmacological inhibition of GPX4 with RSL3 or blockade of system xCT with erastin markedly sensitizes NPC cells to ferroptotic death, underscoring the therapeutic relevance of this pathway [26,44,45].

Beyond these canonical regulators, additional molecules fine-tune this axis in NPC. Thioredoxin-interacting protein (TXNIP) downregulates SLC7A11 and GPX4, thereby increasing ROS accumulation and promoting ferroptosis; its overexpression restores radiosensitivity in resistant cells [31]. Non-coding RNAs also modulate this axis by stabilizing ferroptosis-suppressing transcripts and regulating m^6^A-dependent post-transcriptional programs, collectively enhancing resistance to oxidative stress [46,47]. Pharmacological agents such as erastin, sorafenib, sulfasalazine, and disulfiram/copper complexes additionally target system xCT, reducing cystine import and enhancing ferroptotic stress in NPC preclinical models [40,48]. Collectively, these findings establish the xCT–GSH–GPX4 axis as the core barrier to ferroptosis in NPC.

Among ferroptosis pathways, the xCT–GSH–GPX4 system has the strongest mechanistic and translational support, validated across multiple NPC cell lines, xenografts, and patient-derived datasets. By contrast, several ncRNA-mediated regulators of this axis have been reported only in single-study settings and require further validation in independent cohorts. Overall, these findings establish the xCT–GSH–GPX4 antioxidant system as the central ferroptosis-resistance mechanism in NPC, and its genetic, epigenetic, and metabolic modifiers collectively shape the sensitivity of NPC cells to treatment-induced oxidative stress. Clinically, high SLC7A11 or GPX4 expression in NPC tumors has been associated with poorer survival and reduced response to radiotherapy or platinum-based chemotherapy, underscoring the central role of this axis in determining treatment outcomes.

### 2.2. Iron Metabolism and Ferritinophagy

Iron metabolism is indispensable for ferroptosis, as ferrous iron (Fe^2+^) catalyzes ROS formation via the Fenton reaction, amplifying lipid peroxidation [49]. In NPC, dysregulated ferritinophagy, mediated by nuclear receptor coactivator 4 (NCOA4), increases labile iron pools, rendering cells more susceptible to ferroptosis [21]. Core iron metabolism genes such as transferrin receptor (TFRC), ferroportin (SLC40A1), and ferritin heavy chain (FTH1) have been associated with patient prognosis, and ferroptosis-related gene signatures enriched in iron metabolism strongly predict survival outcomes [37].

Several therapeutic interventions converge on iron metabolism in NPC. Interestingly, itraconazole reduces ferritinophagy-driven iron mobilization, a process on which NPC cells are particularly dependent because of EBV-associated metabolic rewiring [50]. Likewise, cephalosporins enhance HO-1–mediated iron release, a pathway that is constitutively hyperactivated in NPC, thereby producing a more pronounced effect in this tumor type [51]. Conversely, CD38 prevents ferroptosis by stabilizing SLC7A11 through competition with TRIM21, indirectly maintaining antioxidant capacity and enhancing radioresistance [32]. Additional regulators such as the EMC2–TFRC axis and PRMT4-mediated Nrf2/GPX4 signaling further connect iron availability to ferroptosis suppression in NPC [39,52]. These findings emphasize that iron metabolism is a double-edged sword: while excess iron promotes ferroptosis, NPC cells co-opt regulatory mechanisms to restrain iron-dependent oxidative damage and evade death. Targeting ferritinophagy or iron-transport pathways thus represents a promising strategy to resensitize NPC to therapy.

While the role of ferritinophagy and TFRC-driven iron uptake is well supported by converging experimental evidence, other regulators—such as cephalosporin-induced HMOX1 activation or EMC2–TFRC interactions—are based on early-phase observations and should be interpreted cautiously until larger confirmatory studies are available. Taken together, dysregulated iron handling—including ferritinophagy, transferrin signaling, and heme metabolism—creates a dynamic iron pool that can either promote ferroptosis or be suppressed by NPC cells to sustain therapy resistance. Iron metabolism–related gene signatures, including TFRC, FTH1, and SLC40A1, have been shown to stratify patient prognosis and correlate with patterns of immune infiltration, suggesting their utility as clinically relevant biomarkers.

### 2.3. Lipid Metabolism and ACSL4

PUFAs incorporated into phospholipids are essential substrates for ferroptosis. ACSL4 catalyzes the esterification of PUFAs into membrane phospholipids, and its presence determines ferroptotic susceptibility [53,54]. In NPC, ACSL4 acts as a double-edged regulator. Elevated ACSL4 expression supports malignant proliferation, but acetylation of ACSL4 by histone acetyltransferase HAT1 enhances ferroptosis, making NPC cells more radiosensitive [22]. Deacetylation by SIRT3 or regulation by HDAC2 further modulates this process, showing that post-translational modifications of ACSL4 are critical determinants of NPC ferroptotic fate [22]. ACSL4 expression has been linked to ferroptosis sensitivity in multiple cancers, and in NPC specifically, higher ACSL4 levels may mark tumors that are more responsive to radiotherapy due to increased lipid peroxidation susceptibility.

Other lipid-metabolic regulators influence ferroptosis sensitivity. Phosphoenolpyruvate carboxykinase 2 (PCK2) contributes to phospholipid remodeling, and its downregulation in therapy-tolerant tumor-repopulating cells reduces ferroptotic vulnerability, promoting radioresistance [34]. Prolyl 4-hydroxylase subunit alpha 1 (P4HA1) activates the mevalonate pathway through HMGCS1, suppressing ferroptosis and enhancing tumor proliferation [35]. COX-2, often upregulated in NPC, indirectly contributes to ferroptosis modulation by regulating prostaglandins and inflammation, and its inhibition with agents such as celecoxib sensitizes NPC cells to oxidative stress [55]. Thus, lipid metabolism not only provides the substrates for ferroptosis but also represents a site of therapeutic vulnerability. Modulating ACSL4 activity or interfering with metabolic crosstalk, such as PCK2 and P4HA1 pathways, may restore ferroptotic sensitivity in resistant NPC phenotypes.

ACSL4 acetylation as a radiosensitizing mechanism is supported by robust biochemical and in vivo data; however, the contribution of broader lipid metabolic rewiring (e.g., PCK2 or P4HA1–HMGCS1 signaling) to clinical ferroptosis phenotypes in NPC remains more speculative and awaits translational correlation. Emerging lipidomic data suggest that NPC may exhibit a relatively higher PUFA-enriched phospholipid profile compared with other head and neck cancers, although direct comparative studies are still lacking. Thus, lipid metabolic remodeling, particularly via ACSL4 modifications and mevalonate pathway activation, serves as a decisive determinant of ferroptotic vulnerability in NPC.

### 2.4. Epigenetic and Post-Transcriptional Regulation

Epigenetic and post-transcriptional regulators provide another layer of ferroptosis control in NPC. METTL3-mediated m^6^A modification stabilizes SLC7A11 transcripts, thereby preventing radiation-induced ferroptosis and promoting radioresistance [33]. Insulin-like growth factor 2 mRNA-binding protein 2 (IGF2BP2), a m^6^A reader protein, enhances ceruloplasmin stability, restricting iron accumulation and suppressing ferroptosis in NPC cells; high IGF2BP2 expression correlates with poor survival [23].

Additional pathways reinforce ferroptosis evasion. *O*-GlcNAcylated HOXA9 recruits UBR5, which drives SIRT6 degradation, suppressing ferroptosis and promoting tumor progression [24]. NAT10-mediated *N*4-acetylcytidine (ac4C) modification of SLC7A11 further stabilizes this transcript, enabling cisplatin resistance in NPC [40]. FTO, another RNA demethylase, regulates ferroptosis via otubain-1 (OTUB1) stabilization, and aberrant activity of this pathway has been linked to poor therapy response [56].

Non-coding RNAs also modulate ferroptosis. MicroRNAs such as miR-122-5p and long non-coding RNAs including RRFERV influence GPX4 and iron metabolism, thereby indirectly shaping ferroptotic susceptibility [57,58]. CircRNAs like circADARB1 enhance resistance by stabilizing ferroptosis-suppressing transcripts [46]. Together, these epigenetic programs underscore how ferroptosis is not only a metabolic event but also tightly regulated at the RNA and chromatin level in NPC.

Among epigenetic regulators, METTL3 and IGF2BP2 have strong functional and prognostic evidence, whereas newer pathways—including NAT10-ac4C and HOXA9–UBR5–SIRT6 axes—are mechanistically compelling but still require validation in patient-level datasets to establish clinical relevance. Collectively, these epigenetic and post-transcriptional programs protect NPC cells from ferroptosis by stabilizing anti-ferroptotic transcripts and degrading pro-ferroptotic regulators, thereby reinforcing resistance to therapeutic stress. Several of these epigenetic regulators, such as METTL3, IGF2BP2, and HOXA9, exhibit strong associations with recurrence risk and radioresistance in NPC, supporting their potential as prognostic biomarkers and therapeutic targets.

### 2.5. Tumor Microenvironment 

The tumor microenvironment (TME) and EBV infection add further complexity to ferroptosis regulation in NPC. CAFs release FGF5, which activates FGFR2–Nrf2–HO-1 signaling in NPC cells, thereby suppressing cisplatin-induced ferroptosis and fostering chemoresistance [41]. Platelet-derived extracellular vesicles (EVs) transfer integrin β3 (ITGB3) to stabilize SLC7A11, further enhancing ferroptosis resistance and promoting survival under stress [42]. Macrophage M1 polarization also influences ferroptosis; ACSL4-mediated lipid remodeling in macrophages has been linked to inflammatory ferroptotic crosstalk that can either suppress or enhance NPC growth depending on context [59].

EBV, the defining etiologic agent of NPC, directly shapes ferroptosis. EBV-driven activation of Nrf2/Keap1 signaling stabilizes SLC7A11 and GPX4, rendering NPC cells more resistant to ferroptotic stress [25]. Moreover, TNF-α signaling restricts EBV reactivation by modulating GPX4, linking immune control of the virus to ferroptotic signaling [60]. EBV-encoded proteins and microRNAs are increasingly recognized as modulators of iron and lipid metabolism, although their roles in ferroptosis are only beginning to be elucidated.

EBV-driven antioxidant reprogramming is supported by substantial mechanistic and translational evidence, whereas EV-mediated or CAF-derived ferroptosis suppression mechanisms, although intriguing, remain at an earlier developmental stage with limited patient-level validation. Overall, EBV-driven signaling, CAF-derived factors, and EV-mediated communication converge to establish an anti-ferroptotic microenvironment, highlighting the importance of the viral–stromal context in determining ferroptosis sensitivity in NPC. EBV-driven GPX4 stabilization and CAF–FGF5–Nrf2–HO-1 signaling have been linked to chemotherapy resistance in clinical samples, indicating that viral and microenvironmental programs directly contribute to treatment failure.

**Figure 3:**
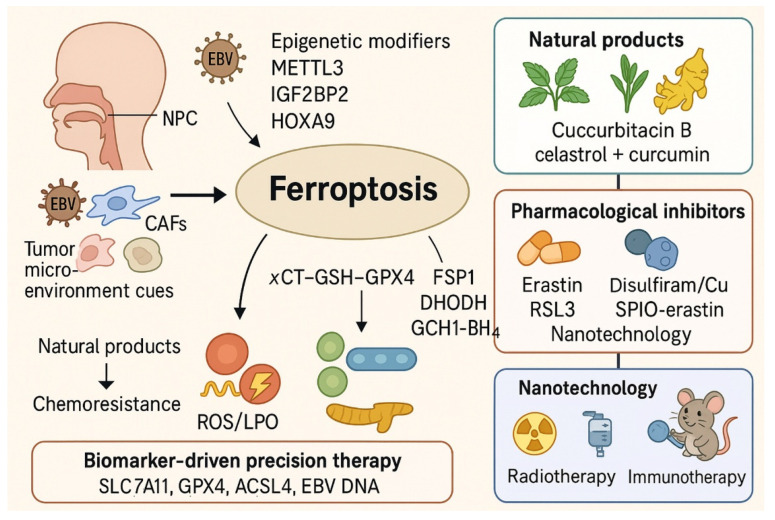
Integrated overview of ferroptosis regulation, therapy resistance, and therapeutic strategies in NPC. This graphical summary illustrates how NPC cells suppress ferroptosis through the xCT–GSH–GPX4 antioxidant axis, epigenetic regulators, EBV-driven signaling, and tumor microenvironmental cues, thereby promoting radioresistance and chemoresistance. Conversely, natural products, GPX4 or HO-1 inhibitors, and nanotechnology-based delivery systems restore ferroptotic vulnerability and enhance the efficacy of radiotherapy, chemotherapy, and immunotherapy. Integration of ferroptosis-targeted interventions with biomarker-based patient stratification offers a precision strategy to overcome therapy resistance in NPC. Abbreviations: ACSL4, acyl-CoA synthetase long-chain family member 4; BH4, tetrahydrobiopterin; CAFs, cancer-associated fibroblasts; Cu, copper; DHODH, dihydroorotate dehydrogenase; EBV, Epstein–Barr virus; FSP1, ferroptosis suppressor protein 1; GCH1, GTP cyclohydrolase 1; GPX4, glutathione peroxidase 4; GSH, glutathione; IGF2BP2, insulin-like growth factor 2 mRNA-binding protein 2; LPO, lipid peroxidation; METTL3, methyltransferase-like 3; NPC, nasopharyngeal carcinoma; ROS, reactive oxygen species; SLC7A11, cystine/glutamate antiporter light chain (xCT); SPIO, superparamagnetic iron oxide; xCT, cystine/glutamate antiporter.

In summary, ferroptosis regulation in NPC is orchestrated by interconnected pathways encompassing antioxidant defenses, iron availability, lipid peroxidation, epigenetic reprogramming, and the tumor-virus-microenvironment axis. Suppression of ferroptosis underlies therapy resistance, while restoration of ferroptosis offers a promising therapeutic route. By targeting key vulnerabilities, such as the system xCT–GSH–GPX4 axis, ferritinophagy, ACSL4 activity, and EBV-driven antioxidant programs, novel strategies may be developed to overcome resistance and improve patient outcomes in NPC. An integrated overview summarizing ferroptosis suppression mechanisms, therapeutic vulnerabilities, and intervention strategies in NPC is illustrated in Figure 3.

## 3. Ferroptosis in NPC Therapy Resistance

Therapy resistance remains a major challenge in the clinical management of NPC. Although RT and chemotherapy remain the mainstay of treatment, 15–20% of patients still develop local recurrence or distant metastasis due to intrinsic or acquired resistance [9,61]. Emerging evidence indicates that dysregulated ferroptosis pathways lie at the heart of both radioresistance and chemoresistance, as NPC cells activate multiple antioxidant and metabolic adaptations to escape iron-dependent oxidative death. Understanding how ferroptosis contributes to therapy response provides a mechanistic framework for designing novel radiosensitizing and chemosensitizing strategies.

### 3.1. Ferroptosis and Radioresistance in NPC

RT eliminates NPC cells primarily through DNA damage and the generation of ROS. Because ferroptosis is characterized by iron-dependent lipid peroxidation, RT-induced ROS has the potential to trigger ferroptosis. However, resistant cells often upregulate ferroptosis-protective pathways, leading to treatment failure [21].

One key mechanism involves the CD38–SLC7A11–GPX4 axis. CD38 stabilizes SLC7A11 by competitively binding to TRIM21, thereby preserving GSH pools and suppressing ferroptosis [32]. CD38 overexpression correlates with enhanced radioresistance in NPC, while silencing CD38 restores ferroptosis and radiosensitivity. Similarly, METTL3-mediated m^6^A modification increases SLC7A11 mRNA stability, preventing RT-induced ferroptosis and promoting resistance [33]. Epigenetic regulation via *O*-GlcNAcylated HOXA9, which recruits UBR5 to degrade SIRT6, further suppresses ferroptosis and enhances RT resistance [24]. These ferroptosis-protective programs not only explain molecular resistance but also reflect clinical behavior, as patients with high SLC7A11, GPX4, METTL3, or CD38 expression frequently demonstrate inferior radiotherapy response and shorter progression-free survival.

In contrast, several regulators sensitize NPC to RT by enhancing ferroptosis. Acetylation of ACSL4, mediated by HAT1/HDAC2, stabilizes ACSL4 protein and promotes ferroptotic lipid peroxidation, thereby increasing radiosensitivity [22]. Downregulation of PCK2, observed in radiation-tolerant NPC cells, reduces ferroptosis and augments DNA repair, while restoration of PCK2 expression and phospholipid remodeling re-sensitizes cells to RT [34,62]. TXNIP acts as a radiosensitizer by downregulating SLC7A11 and GPX4; its overexpression promotes ROS accumulation and ferroptotic death in a dose-dependent manner [31]. Additionally, lncRNA RRFERV enhances TEAD1 signaling to stabilize ferroptosis regulators, conferring radioresistance but simultaneously creating vulnerability to ferroptosis inducers, providing a therapeutic entry point [57]. Glutathione *S*-transferase mu 3 (GSTM3) promoted RT-induced ferroptosis and enhanced radiosensitivity of NPC via USP14/FASN and GPX4 axes [63].

The tumor microenvironment also shapes ferroptosis-mediated RT response. Local angiotensin II signaling activates the HIF-1α–HILPDA pathway, suppressing ferroptosis and lipid peroxidation, thereby reducing radiosensitivity [64]. Pharmacological blockade of angiotensin II signaling with ARBs restores ferroptosis and enhances RT efficacy. Likewise, depletion of superoxide dismutase 2 (SOD2), a mitochondrial antioxidant enzyme, sensitizes NPC cells to RT by enhancing ferroptosis, but this effect can be reversed by DHODH inhibition, cautioning the clinical use of DHODH inhibitors during RT [65]. Additionally, the CAPRIN2–HMGCR axis, regulated by upstream LINC00941, increased the ferroptosis resistance and survival of extracellular matrix-detached tumor cells, which contributed to NPC metastatic colonization [66].

Collectively, ferroptosis is both a mediator and a modulator of RT response. Resistance arises when NPC cells stabilize GPX4 and SLC7A11 or activate adaptive antioxidant pathways, while targeting these mechanisms through TXNIP induction, ACSL4 acetylation, or PCK2 restoration can resensitize tumors to irradiation.

### 3.2. Ferroptosis and Chemoresistance in NPC

Platinum-based agents such as cisplatin, as well as taxanes, remain essential in NPC treatment. However, drug resistance severely limits efficacy, and ferroptosis suppression has emerged as a key contributor. HO-1 is strongly induced by cisplatin and confers resistance by reducing oxidative stress and inhibiting ferroptosis. Pharmacological inhibition of HO-1 with zinc protoporphyrin (ZnPP) or induction of ferroptosis with erastin restores cisplatin sensitivity in resistant cells, both in vitro and in xenograft models [36]. In the tumor microenvironment, CAFs secrete FGF5, which activates FGFR2–Nrf2–HO-1 signaling, thereby reducing cisplatin-induced ferroptosis and contributing to resistance [41]. However, systemic HO-1 inhibition carries potential toxicity risks, including exacerbation of oxidative or inflammatory injury in normal tissues, and will require carefully controlled, tumor-targeted delivery strategies for safe clinical application.

Other mechanisms include UCHL1–CAV1 stabilization, which inhibits ferroptosis and promotes docetaxel resistance. Silencing UCHL1 or treating with erastin enhances docetaxel efficacy, underscoring the therapeutic potential of targeting this axis [67]. NAT10-mediated ac4C modification of SLC7A11 enhances sorafenib resistance by preventing ferroptosis; inhibiting NAT10 restores sorafenib sensitivity [40]. Likewise, protein arginine methyltransferase 4 (PRMT4) upregulation promotes cisplatin resistance by stabilizing Nrf2 and activating the GPX4 pathway, while PRMT4 knockdown restores ferroptotic sensitivity [39].

Viral oncogenesis further contributes to ferroptosis evasion. EBV infection induces GPX4 stabilization via p62–Keap1–Nrf2 signaling, promoting chemoresistance and tumor progression [25]. EBV reactivation may be inhibited by TNF-α via the GSH–GPX4 axis [60]. Knockdown of GPX4 or pharmacological inhibition restores drug sensitivity in EBV-positive NPC cells [25]. Hypoxia also enhances ferroptotic resistance by inducing BAP1 stabilization of H2A, which suppresses SLC7A11 expression but paradoxically sensitizes cells to ferroptosis under erastin treatment, offering context-dependent vulnerabilities [38]. Because EBV-negative NPC lacks viral oncoprotein–driven Nrf2 activation, the GPX4-stabilizing mechanism described here is largely restricted to EBV-positive disease. EBV-negative tumors may still upregulate GPX4 through alternative, EBV-independent stress-response pathways, but direct evidence for the same stabilization process remains limited.

On the other hand, ferroptosis induction has shown promise in reversing chemoresistance. Berberine inhibits the system xCT–GSH–GPX4 axis, reducing GSH and GPX4 levels, increasing lipid ROS, and suppressing metastasis in NPC models [26]. Cucurbitacin B induces ferroptotic death by downregulating GPX4 and depleting GSH, enhancing cisplatin efficacy in xenograft models [27]. Solasodine triggers ferroptosis by upregulating HMOX1 while suppressing GPX4 and SLC40A1, markedly inhibiting tumor growth [28]. Celastrol-curcumin combination therapy further enhances ferroptosis by upregulating ACSL4 and downregulating GPX4/SLC7A11, leading to synergistic cytotoxicity with conventional chemotherapy [29].

Conversely, tumors with elevated HO-1, UCHL1, NAT10, or EBV-driven antioxidant signatures are consistently enriched among clinical cases of cisplatin or taxane resistance. Taken together, ferroptosis suppression is a consistent feature of therapy-resistant NPC. However, only a subset of pathways—such as SLC7A11/GPX4, METTL3, CD38, and HO-1—are supported by strong mechanistic and clinical associations, whereas several others are based on early-phase findings that need broader validation before being considered therapeutic targets.

In summary, both radioresistance and chemoresistance in NPC are tightly linked to ferroptosis dysregulation. Oncogenes, viral proteins, and epigenetic modifiers converge to suppress ferroptotic pathways, thereby enabling tumor survival under therapeutic pressure. Conversely, restoration of ferroptosis through TXNIP induction, ACSL4 acetylation, HO-1 inhibition, or pharmacological agents such as erastin, berberine, or solasodine offers promising avenues to overcome resistance. Ultimately, ferroptosis represents a central determinant of therapy response in NPC, and its targeted modulation could transform treatment paradigms by resensitizing resistant tumors and improving clinical outcomes.

## 4. Natural Compounds and Pharmacological Agents Inducing Ferroptosis in NPC

Induction of ferroptosis has gained increasing attention as a therapeutic strategy to overcome resistance in NPC. Mechanistically, agents that induce ferroptosis in NPC function by targeting the system xCT–GSH–GPX4 antioxidant axis, disrupting iron metabolism and lipid homeostasis, or inhibiting intrinsic anti-ferroptotic defenses (Table 2). Additionally, nanotechnology-based delivery systems have been employed to amplify oxidative stress in a spatially and temporally controlled manner, highlighting translational potential [20,68].

### 4.1. Canonical Ferroptosis Activators as Mechanistic Tools

Small molecules such as erastin and RSL3 remain the prototypical ferroptosis inducers and are widely used as mechanistic “positive controls.” Erastin inhibits system xCT, reducing cystine uptake and depleting intracellular GSH, while RSL3 directly inactivates GPX4, thereby preventing the detoxification of lipid peroxides. In NPC models, both compounds have been used to demonstrate that blockade of the cystine–GSH–GPX4 axis sensitizes tumor cells to ferroptotic death, particularly in GPX4-low or xCT-addicted phenotypes [26,79]. Other small molecules with similar activity have been explored. Sulfasalazine, an FDA-approved drug, has been reported to inhibit xCT and mimic the effects of erastin, providing a clinically relevant scaffold for ferroptosis-based interventions [70,71]. Sorafenib, a multi-kinase inhibitor, also possesses ferroptosis-inducing activity through suppression of system xCT and depletion of GSH, and has been tested in NPC cells as an adjunct to radiation and chemotherapy [40,72]. Disulfiram, a drug used in alcohol dependence, forms complexes with copper (DSF/Cu) that disrupt cellular redox homeostasis, deplete GSH, and trigger lipid ROS accumulation, making it another example of a repurposed agent with ferroptotic potential in NPC [48]. Together, these canonical activators provide mechanistic insights into ferroptosis regulation in NPC and serve as reference compounds for the development of novel therapeutic candidates.

### 4.2. Natural Products as Ferroptosis Inducers

Natural products constitute a rich source of bioactive compounds capable of modulating ferroptosis in NPC. Solasodine, a steroidal alkaloid, was shown to suppress NPC progression by inducing mitochondrial damage, elevating Fe^2+^, ROS, and malondialdehyde (MDA) levels, and depleting GSH [28]. This process coincided with downregulation of GPX4 and SLC40A1 and upregulation of COX-2 and HO-1. Importantly, ferroptosis inhibitors but not apoptosis inhibitors reversed solasodine-induced cytotoxicity, confirming ferroptosis as the dominant mode of cell death. Another well-studied example is the combination of celastrol and curcumin. While celastrol alone exhibited limited cytotoxicity in NPC cells, its combination with curcumin markedly enhanced ferroptotic death through ACSL4 upregulation and suppression of GPX4 and SLC7A11 [29]. In xenograft models, this combination achieved potent tumor inhibition with minimal systemic toxicity, underscoring the therapeutic promise of combinatorial natural products.

Beyond these, multiple other natural compounds have demonstrated ferroptosis-inducing effects in NPC. Berberine exerts anti-proliferative and anti-metastatic effects by inhibiting the xCT–GSH–GPX4 axis, leading to GSH depletion, lipid peroxidation, and ROS accumulation [26]. Cucurbitacin B induces ferroptosis by increasing intracellular iron and downregulating GPX4, thereby sensitizing NPC cells to chemotherapy [27]. Luteolin enhances ferroptotic sensitivity through suppression of the SOX4/GDF15 pathway, resulting in decreased GPX4 and GSH levels and increased lipid ROS [73]. Luteolin exhibits antioxidant and anticancer activities relevant to ferroptosis regulation [80]. Isoquercitrin promotes ferroptosis via AMPK/NF-κB signaling, inducing oxidative stress and tumor suppression both in vitro and in xenograft models [74]. Allicin, derived from garlic, has also been reported to suppress NPC proliferation by decreasing GPX4 and GSH while enhancing lipid ROS, supporting the potential of dietary compounds in ferroptosis-based therapy [75]. Lupeol, a triterpenoid found in fruits and vegetables, has been shown to potentiate ferroptosis by inhibiting GPX4 activity and destabilizing redox balance, further contributing to anti-tumor effects in NPC [76]. Collectively, these natural products provide a diverse chemical arsenal that can be harnessed to induce ferroptosis and complement conventional therapies in NPC.

### 4.3. Pharmacologic Modulation of Ferroptosis

Pharmacological modulation of ferroptosis in NPC extends beyond canonical inducers and natural products, encompassing drugs that directly or indirectly regulate anti-ferroptotic defenses. Cisplatin-resistant NPC cells, for example, exhibit elevated HO-1, which attenuates oxidative stress and blocks ferroptosis [36]. Pharmacologic inhibition of HO-1 with ZnPP, or combination with erastin, restores cisplatin sensitivity and suppresses tumor growth in xenografts. Another pathway involves UCHL1, a deubiquitinating enzyme that stabilizes caveolin-1 (CAV1). UCHL1–CAV1 interaction suppresses lipid peroxidation and ferroptosis in NPC cells, conferring resistance to docetaxel [67]. Silencing UCHL1 or pharmacologically inhibiting this axis restores ferroptotic activity and enhances docetaxel efficacy.

Beyond these, other pharmacologic interventions have been explored. Cephalosporin antibiotics, surprisingly, were found to selectively induce ferroptosis in NPC cells via HMOX1 activation, suggesting drug repurposing as a practical avenue for ferroptosis-based therapy [51]. PRMT4 has been identified as an upstream regulator of the Nrf2/GPX4 pathway; its overexpression enhances resistance to cisplatin, while PRMT4 inhibition restores ferroptotic sensitivity [39]. NAT10, an RNA acetyltransferase, promotes chemoresistance by stabilizing SLC7A11 through *N*4-acetylcytidine modification, but its inhibition reduces xCT expression and sensitizes cells to ferroptosis-inducing drugs [40]. Moreover, FSP1, a ferroptosis suppressor acting independently of GPX4, has been implicated in NPC, and preclinical inhibitors such as iFSP1 have been shown to suppress the FSP1–CoQ_10_ axis and sensitize cancer cells to ferroptosis-inducing therapies [20,78].

### 4.4. Nanotechnology-Enabled Ferroptosis

Nanotechnology has opened new avenues for ferroptosis-based therapy in NPC by allowing targeted delivery and controlled release of ferroptosis inducers. A gelatin-alginate hydrogel incorporating Bi_2_Se_3_ nanosheets was engineered to release ROS upon near-infrared activation. This platform induced ferroptosis and apoptosis simultaneously, ablated NPC tumors, and exhibited anti-inflammatory and tissue-healing effects that reduced collateral damage [30]. Other designs, such as superparamagnetic iron oxide nanoparticles loaded with erastin, enable combined diagnostic imaging and therapeutic ferroptosis induction [77]. Nanocarriers targeting ferroptosis-resistance drivers, such as circADARB1, have also been developed to enhance radiosensitivity and promote ferroptotic cell death in resistant NPC models [46]. However, these nanoplatforms also face important limitations, including restricted penetration into the dense stromal and hypoxic regions of NPC and rapid immune clearance by the mononuclear phagocyte system, which may reduce intratumoral accumulation. Addressing these barriers through surface modification, size optimization, or immune-evasive coatings will be essential for successful clinical translation.

### 4.5. Practical Considerations for Combination Therapy

Ferroptosis induction has demonstrated synergy with radiotherapy and immunotherapy. Radiotherapy inherently produces ROS and lipid radicals, and ferroptosis induction magnifies this oxidative stress, enhancing radiosensitivity. Furthermore, ferroptotic death releases lipid peroxidation-derived signals and damage-associated molecular patterns (DAMPs), augmenting immunogenicity and potentially improving checkpoint blockade efficacy. Preclinical data support the integration of ferroptosis inducers with PD-1/PD-L1 inhibitors or RT to achieve durable responses [4,8]. However, challenges remain, including the limited bioavailability and potential toxicity of natural compounds. Advances in formulation strategies such as liposomes, nanoparticles, and hydrogels are critical to improving delivery and minimizing off-target effects, paving the way for clinical translation.

In summary, natural compounds such as solasodine, berberine, cucurbitacin B, luteolin, isoquercitrin, allicin, and celastrol-curcumin combinations, pharmacological modulators including ZnPP, disulfiram/copper complexes, and cephalosporins, and nanotechnology-based platforms converge on disrupting the SLC7A11–GPX4 axis and enhancing iron-driven lipid peroxidation. By re-sensitizing NPC cells to radiotherapy and chemotherapy, ferroptosis induction offers a robust strategy for overcoming therapy resistance and advancing toward clinical translation.

Most ferroptosis-inducing agents discussed in this section—including natural products, HO-1 inhibitors, GPX4 antagonists, and nanotechnology-based carriers—remain at the preclinical stage with no clinical trials yet conducted in NPC. Accordingly, their therapeutic potential should be interpreted cautiously until safety, pharmacokinetics, and efficacy are evaluated in early-phase human studies. Although many natural compounds demonstrate ferroptosis-inducing activity in vitro and in xenografts, their pharmacologic relevance remains uncertain due to limited bioavailability, lack of toxicity profiling, and the absence of clinical data, underscoring the speculative nature of these findings.

## 5. Biomarkers and Patient Stratification for Ferroptosis-Targeted Therapy in NPC

Biomarkers are central to translating ferroptosis research into clinical practice, as they enable the identification of patients most likely to benefit from ferroptosis-targeted interventions. In NPC, ferroptosis susceptibility is shaped by genetic determinants, epigenetic reprogramming, viral infection, and the tumor microenvironment (Table 3). A comprehensive biomarker framework that integrates these layers offers a path toward precision stratification and adaptive therapy in clinical settings.

### 5.1. Ferroptosis-Related Genes and Prognostic Signatures

Ferroptosis-related genes (FRGs) such as SLC7A11, GPX4, ACSL4, TFRC, and FTH1 have been repeatedly highlighted as central to ferroptosis regulation in NPC. Elevated SLC7A11 expression enhances cystine uptake and GSH biosynthesis, conferring protection from lipid peroxidation and correlating with radioresistance and poor survival [32,33,44]. Similarly, GPX4, a major ferroptosis suppressor, is highly expressed in therapy-resistant NPC and is associated with adverse prognosis [31,39,63]. By contrast, ACSL4, which drives PUFA enrichment in membranes, confers vulnerability to ferroptosis and may act as a positive predictor of radiosensitivity [22]. A key immune invasion-related ferroptosis-related factor, ATG5, is highly expressed in the NPC and head and neck cancer patients, related to poor survival outcomes after immune checkpoint blockade therapy [85]. Large-scale transcriptomic studies using weighted gene co-expression network analysis (WGCNA) have identified hub genes such as TBK1, KIF20A, SLC16A1, and QSOX1, which not only stratify patient outcomes but also predict ferroptosis activity in NPC tumors [37]. Integrating iron metabolism-related gene signature (IMRGs) has revealed TFRC, SLC39A14, and ATP6V0D1, which are associated with prognosis and immune infiltration in NPC patients [81]. These findings highlight ferroptosis-related gene panels as potential prognostic biomarkers for patient stratification.

### 5.2. Epigenetic and Post-Transcriptional Biomarkers

Epigenetic and post-transcriptional modifications provide another layer of regulatory control over ferroptosis and are increasingly recognized as biomarkers. METTL3, an m6A methyltransferase, stabilizes SLC7A11 mRNA through *N*6-methyladenosine modification, thereby suppressing ferroptosis and promoting radioresistance [33]. In parallel, the m^6^A reader IGF2BP2 stabilizes ceruloplasmin mRNA, reducing iron overload and lipid peroxidation, and high IGF2BP2 levels correlate with poor prognosis [23]. Moreover, *O*-GlcNAcylated HOXA9 recruits UBR5 to degrade SIRT6, further restraining ferroptosis and driving NPC progression [24]. Other RNA regulators, such as lncRNA HOTAIRM1 and circADARB1, modulate ferroptosis resistance through m^6^A-dependent and proteostasis-linked mechanisms, indicating their potential as predictive biomarkers for therapy response [46,47]. These epigenetic biomarkers collectively expand the scope of stratification beyond genetic mutations, incorporating transcriptomic and post-transcriptional landscapes.

### 5.3. Tumor Microenvironment and Viral Factors

The TME and EBV infection strongly influence ferroptosis in NPC. EBV activates the Nrf2/Keap1–SLC7A11/GPX4 axis, enhancing antioxidant defenses and suppressing ferroptosis, which contributes to both tumor progression and therapy resistance [25,60]. Moreover, TME-derived factors such as CAF-secreted FGF5, which activates FGFR2 and downstream Nrf2–HO-1 signaling, inhibit cisplatin-induced ferroptosis and reduce chemotherapy efficacy [41]. Platelet-derived EVs carrying ITGB3 also stabilize SLC7A11, suppress ferroptosis, and promote metastasis [42]. Immune infiltration patterns provide additional biomarker potential: tumors with higher ferroptosis-related gene expression demonstrate increased infiltration of M1 macrophages and neutrophils, indicating that immune signatures may reflect ferroptotic activity [59,82,86]. Finally, plasma EBV DNA, an established prognostic marker in NPC, may indirectly capture ferroptosis dynamics by reflecting tumor metabolic and oxidative stress states [4].

### 5.4. On-Treatment Biomarkers for Dynamic Stratification

Static biomarkers provide baseline prediction, but dynamic monitoring is critical to assess real-time ferroptosis induction during therapy. Experimental models demonstrate that ferroptosis can be tracked by reductions in intracellular GSH, increases in MDA as a lipid peroxidation byproduct, and elevated Fe^2+^ pools [83]. Integration of these metabolic indicators with established clinical tools, such as circulating EBV DNA kinetics, enables dynamic stratification of patients during radiotherapy or chemotherapy [4]. Advances in liquid biopsy technologies may further allow detection of ferroptosis-related metabolites and extracellular vesicle cargo in patient plasma, improving real-time monitoring of treatment efficacy [84,87].

### 5.5. Toward Precision Stratification

Collectively, ferroptosis biomarkers in NPC span genetic, epigenetic, viral, immune, and metabolic domains. Baseline profiling of FRGs such as SLC7A11, GPX4, ACSL4, and TFRC can identify patients at higher risk of treatment resistance, while epigenetic regulators including METTL3, IGF2BP2, HOXA9, and NAT10 refine prognostic stratification. Integration of viral status (EBV DNA load) and immune infiltration patterns provides additional context for ferroptotic vulnerability. Finally, on-treatment biomarkers such as GSH depletion, MDA accumulation, and Fe^2+^ elevation allow dynamic adaptation of therapy. This multilayered biomarker framework underscores that ferroptosis-targeted strategies in NPC are most likely to succeed when guided by comprehensive, biomarker-driven patient selection. Precision stratification, integrating molecular, viral, immune, and metabolic cues, will be essential to translate ferroptosis from bench to bedside in NPC management. Collectively, these biomarker studies highlight that ferroptosis regulators are not merely mechanistic molecules but also clinically meaningful predictors of prognosis, treatment sensitivity, and resistance patterns in NPC.

## 6. Therapeutic Implications

The expanding body of evidence linking ferroptosis to NPC biology highlights its potential as a novel therapeutic avenue. By integrating ferroptosis modulation with established modalities such as radiotherapy, chemotherapy, and immunotherapy, and by targeting intrinsic anti-ferroptotic defenses, it may be possible to overcome treatment resistance and significantly improve patient outcomes (Table 4). Translating ferroptosis into the clinic will require a deeper understanding of mechanistic interactions, optimized delivery systems, and comprehensive biomarker-guided stratification.

### 6.1. Ferroptosis-Based Radiosensitization

RT is the standard treatment for NPC, yet the development of radioresistance remains a major clinical obstacle [89]. Ionizing radiation generates DNA damage as well as lipid peroxidation and iron-mediated ROS, all of which can trigger ferroptosis [90]. In resistant cells, ferroptosis is suppressed through adaptive mechanisms. For instance, CD38 stabilizes SLC7A11, maintaining GSH pools and preventing ferroptosis, while METTL3-mediated m^6^A modification of SLC7A11 further enhances radioresistance [32,33]. HOXA9–UBR5–SIRT6 signaling suppresses ferroptosis through epigenetic modulation, reinforcing survival under radiation stress [24].

Conversely, regulators such as acetylated ACSL4 or TXNIP upregulation promote ferroptosis and radiosensitize NPC cells by amplifying ROS and lipid peroxidation [22,31]. Restoration of PCK2 expression in radio-tolerant tumor cells has also been shown to resensitize them by facilitating ferroptotic cell death [34]. These insights suggest that combining ferroptosis inducers (erastin, RSL3, or natural products) with RT could improve tumor control and reduce relapse rates by tipping the oxidative balance toward ferroptosis [21,71]. Although ferroptosis-based radiosensitization shows strong mechanistic rationale and robust preclinical activity, no clinical studies have yet tested ferroptosis inducers with radiotherapy in NPC, highlighting a critical evidence gap.

### 6.2. Ferroptosis in Overcoming Chemoresistance

Platinum-based regimens such as cisplatin remain central to NPC therapy, yet resistance is common. Ferroptosis is a key determinant of chemosensitivity. Cisplatin-resistant NPC cells often upregulate HO-1, which degrades pro-oxidant heme and attenuates ferroptosis [36]. Inhibiting HO-1 with ZnPP or combining with erastin restores cisplatin efficacy in vitro and in vivo. Similarly, the UCHL1-CAV1 axis prevents ferroptosis and promotes docetaxel resistance, but silencing UCHL1 re-sensitizes cells and enhances chemotherapy responses [67].

Natural compounds also act as chemosensitizers by promoting ferroptosis. Solasodine enhances cisplatin cytotoxicity by inducing lipid peroxidation and suppressing GPX4, while berberine and cucurbitacin B decrease GPX4/SLC7A11 levels, facilitating drug sensitivity [26,27,28]. Celastrol combined with curcumin markedly enhances ferroptotic death and potentiates cisplatin response in xenografts with minimal toxicity [29]. These examples demonstrate that targeting ferroptosis can directly reverse chemoresistance, especially when coupled with standard drugs. Among the pathways described, only HO-1 expression and baseline EBV DNA dynamics have been explored in clinical settings, whereas most ferroptosis-targeted chemosensitizers remain supported exclusively by preclinical models.

### 6.3. Ferroptosis and Immunotherapy Synergy

Immunotherapy, particularly PD-1 blockade, has transformed treatment for recurrent or metastatic NPC, though response rates remain limited [8]. Ferroptosis is inherently immunogenic: lipid peroxidation products such as 4-hydroxynonenal (4-HNE) and oxidized phospholipids activate dendritic cells and promote T-cell priming [91]. Ferroptotic death also enhances antigen release, increasing tumor immunogenicity.

Preclinical studies indicate that ferroptosis inducers convert the NPC microenvironment into a pro-inflammatory milieu, thereby amplifying checkpoint inhibitor responses [20]. Moreover, strategies that propagate ferroptosis to resistant tumor subpopulations, including cancer stem-like cells, may prevent immune evasion [92,93]. These findings support the rationale for combining ferroptosis inducers with PD-1/PD-L1 inhibitors to improve immunotherapy outcomes in NPC [88]. At present, ferroptosis–immunotherapy combinations are entirely conceptual in NPC and supported only by early mechanistic findings from other tumor types, underscoring the need for well-designed translational studies.

### 6.4. Targeting Anti-Ferroptosis Pathways

NPC cells deploy multiple anti-ferroptotic pathways to evade oxidative death. These include the FSP1–CoQ_10_–vitamin K axis, which regenerates lipid antioxidants [94,95]; HO-1 upregulation, which prevents cisplatin-induced ferroptosis [36]; and the UCHL1–CAV1 pathway, which stabilizes membrane structure and blocks lipid ROS accumulation [67]. Epigenetic programs further contribute, with METTL3, IGF2BP2, and HOXA9 all acting as ferroptosis suppressors [23,24,33]. Targeting these “anti-ferroptosis” defenses may sensitize NPC cells to therapy. For example, blocking UCHL1 or HO-1 restores chemosensitivity, while inhibitors of METTL3 or NAT10 may counteract epigenetic ferroptosis resistance. Thus, the therapeutic strategy is not only to induce ferroptosis but also to neutralize anti-ferroptotic pathways that NPC cells exploit for survival.

### 6.5. Nanotechnology and Drug Delivery Systems

Many ferroptosis inducers face challenges of poor bioavailability and systemic toxicity. Nanotechnology provides innovative solutions for effective delivery. For instance, Bi_2_Se_3_ nanosheet–alginate hydrogels release ROS upon near-infrared activation, simultaneously inducing ferroptosis and apoptosis, ablating NPC tumors, and promoting local tissue healing [30]. Liposomal formulations of curcumin or erastin analogs improve solubility and tumor penetration, while iron oxide-based nanoparticles facilitate ferroptosis induction coupled with imaging capabilities [77]. Nanocarriers targeting circRNAs or epigenetic regulators further enhance radiosensitization and overcome therapy resistance [46]. Collectively, these nanotechnology-enabled systems expand the therapeutic window and support the clinical feasibility of ferroptosis-based interventions.

### 6.6. Clinical Translation and Challenges

Despite strong preclinical evidence, significant barriers must be addressed before ferroptosis-targeted strategies enter routine clinical practice. First, normal tissue toxicity remains a concern, as ferroptosis may inadvertently damage organs sensitive to oxidative stress, including the heart and liver [96,97]. Second, inter-patient heterogeneity in ferroptosis gene expression, EBV-driven signaling, and metabolic profiles necessitates biomarker-guided patient selection [37,98,99]. Third, the safety of combining ferroptosis inducers with RT or immunotherapy is not yet established, and the risk of synergistic toxicity must be carefully evaluated [100,101]. Finally, the lack of clinically approved ferroptosis-specific drugs limits translation; most agents are either experimental (erastin, RSL3) or repurposed (sulfasalazine, disulfiram) [102]. Overall, only a small subset of ferroptosis-related biomarkers (e.g., SLC7A11, GPX4, EBV DNA kinetics) have been correlated with clinical outcomes in NPC, and none of the therapeutic combinations described have progressed into clinical testing, reflecting a substantial gap between mechanistic insights and clinical translation.

Nonetheless, ferroptosis offers a compelling therapeutic opportunity for NPC. Integration with multimodal therapies, supported by dynamic biomarkers such as GSH depletion, MDA accumulation, Fe^2+^ levels, and EBV DNA kinetics, may enable adaptive treatment approaches that maximize efficacy while minimizing risk. It is important to distinguish that while ferroptosis-based radiosensitization and HO-1 or GPX4 targeting strategies are supported by substantial preclinical data, clinical translation of nanoparticles, circRNA-targeting platforms, and combination regimens remains at a conceptual stage. Carefully designed early-phase clinical trials are needed to define dosing, timing, and patient stratification strategies. With these efforts, ferroptosis could emerge as a cornerstone of precision medicine in NPC management.

A practical roadmap for clinical translation may include: (1) early-phase window-of-opportunity trials testing ferroptosis-inducing agents with real-time monitoring of dynamic biomarkers such as GSH depletion, MDA accumulation, and EBV DNA kinetics; (2) biomarker-stratified patient selection based on SLC7A11, GPX4, ACSL4, or iron metabolism signatures; (3) phase I/II combination trials integrating low-dose ferroptosis inducers with radiotherapy or platinum doublets to evaluate safety, dose-limiting toxicities, and preliminary efficacy; and (4) adaptive trial designs incorporating pharmacodynamic endpoints to identify optimal timing and sequencing. Such structured steps provide a feasible strategy for moving ferroptosis-targeted therapies from preclinical promise into clinical practice.

### 6.7. Risk–Benefit Considerations and Translational Barriers

Although ferroptosis-based therapies hold substantial promise, their clinical implementation requires careful evaluation of organ-specific risks, delivery challenges, and regulatory barriers. Ferroptosis is intrinsically linked to iron metabolism and oxidative stress, making the liver, kidneys, and myocardium particularly susceptible to unintended injury. Preclinical studies demonstrate that excessive ferroptotic activation can exacerbate hepatic inflammation, promote renal tubular dysfunction, and impair cardiomyocyte viability, highlighting the need for controlled induction and precise targeting.

Mitigation strategies include spatially confined delivery platforms such as near-infrared–activated hydrogels, superparamagnetic nanoparticles, and intratumoral injections, as well as the potential use of protective agents that shield normal tissues without attenuating tumor-specific ferroptosis. These strategies aim to widen the therapeutic window while limiting systemic toxicity.

Beyond biologic considerations, significant translational barriers remain. Few ferroptosis inducers have undergone GMP-standardization or safety profiling suitable for regulatory submission, and no validated pharmacodynamic biomarkers exist for clinical monitoring. Additional limitations include poor pharmacokinetic properties, rapid metabolic degradation, and lack of tumor selectivity for many agents currently used in preclinical studies. Ultimately, overcoming these obstacles will require coordinated integration of nanotechnology, biomarker science, and rational trial design to ensure safe and effective clinical translation of ferroptosis-targeted therapies.

## 7. Conclusions and Perspectives

Ferroptosis represents a paradigm shift in our understanding of cell death in NPC. It has become evident that ferroptosis is intimately linked to radioresistance, chemoresistance, and the complex interplay between tumor cells, EBV infection, and the immune microenvironment. Experimental findings consistently demonstrate that modulating ferroptosis can sensitize NPC cells to conventional therapies and suppress malignant progression, positioning ferroptosis as a promising therapeutic target [4,20]

Despite these advances, important limitations remain. Most current studies rely on in vitro or xenograft models, which only partially capture the complexity of the NPC tumor microenvironment. The heterogeneity of NPC, shaped by viral biology, genetic background, and metabolic context, suggests that ferroptosis-targeted interventions may not benefit all patients equally. Furthermore, excessive or uncontrolled ferroptosis could damage normal tissues, particularly those sensitive to oxidative stress, underscoring the need for selective and context-specific therapeutic approaches [13].

Future research must therefore address several critical priorities. These include mapping ferroptosis regulators across NPC clinical subtypes, clarifying the precise contribution of EBV-driven signaling pathways, and dissecting how ferroptosis interacts with DNA damage repair, hypoxia, and tumor immunity. Biomarker development and validation remain central. Integrating ferroptosis-related gene expression, epigenetic regulators, immune infiltration patterns, and plasma EBV DNA into clinically actionable frameworks will be essential for precision stratification. Real-time monitoring of ferroptosis using dynamic biomarkers such as glutathione depletion, malondialdehyde accumulation, and circulating redox metabolites should also be incorporated into clinical trial design.

Therapeutically, combining ferroptosis inducers with radiotherapy, chemotherapy, or immunotherapy offers synergistic opportunities, but optimal dosing regimens, sequencing strategies, and safety profiles remain undefined. Advances in nanotechnology and drug delivery systems hold promise for enhancing efficacy and minimizing toxicity, yet these approaches require rigorous preclinical and clinical validation. Importantly, ferroptosis-based therapies must be developed with a balanced perspective: while their induction may overcome therapy resistance, controlled inhibition of ferroptosis might also be beneficial in protecting normal tissues or mitigating treatment-associated toxicities.

In summary, ferroptosis is emerging as both a therapeutic vulnerability and an opportunity in NPC. Moving forward, multidisciplinary collaboration across oncology, virology, immunology, and nanotechnology will be required to translate mechanistic insights into durable clinical benefit. As several ferroptosis-regulatory pathways vary widely in evidentiary strength, future studies must prioritize validating the most robust targets (e.g., SLC7A11, GPX4, METTL3, HO-1) in clinical cohorts while critically examining more speculative mechanisms. With continued efforts in biomarker science, drug development, and clinical trial design, ferroptosis-targeted strategies have the potential to transform the management of NPC and address one of its most persistent challenges—therapy resistance.

## Figures and Tables

**Table 1:** Mechanistic axes of ferroptosis in NPC and therapeutic relevance.

Axis	Key Regulator(s) in NPC	Direction on Ferroptosis	Phenotypic Impact in NPC	Model(s)	Reference(s)
System xCT–GSH–GPX4	SLC7A11, GPX4	Anti-ferroptosis	Enhances radio/chemoresistance via ROS detoxification	Multiple NPC cell lines; patient samples	[20,21]
System xCT–ROS linkage	TXNIP ↓ in resistant NPC	Pro-ferroptosis	Overexpression restores radiosensitivity by suppressing xCT–GSH–GPX4 axis	Patient samples; xenografts	[31]
Ubiquitin control of xCT	CD38–TRIM21–SLC7A11	Anti-ferroptosis	Stabilizes SLC7A11; confers radioresistance	In vitro NPC; co-immunoprecipitation validation	[32]
Epigenetic m^6^A regulation	METTL3–SLC7A11; IGF2BP2–CP	Anti-ferroptosis	m^6^A stabilization of SLC7A11 and CP suppresses ferroptosis, promotes radioresistance and progression	NPC lines; xenografts	[23,33]
Epigenetic post-translational control	HOXA9 (O-GlcNAcylated)–UBR5–SIRT6	Anti-ferroptosis	SIRT6 degradation restrains ferroptosis; enhances RT resistance	NPC cells; xenografts	[24]
Lipid metabolism	ACSL4 (PUFA enrichment); ACSL4 acetylation; PCK2; P4HA1–HMGCS1 axis	ACSL4 acetylation: Pro-ferroptosis; PCK2 ↓ and P4HA1 ↑: Anti-ferroptosis	ACSL4 acetylation restores radiosensitivity; PCK2 downregulation suppresses ferroptosis; P4HA1 promotes proliferation	NPC lines; xenograft	[22,34,35]
Iron/heme stress	HO-1 (HMOX1); ferritinophagy (NCOA4); TFRC, FTH1, SLC40A1	HO-1: Anti-ferroptosis; TFRC/NCOA4: Pro-ferroptosis	HO-1 upregulation confers cisplatin resistance; ZnPP inhibition restores ferroptosis	HK1, C666-1; xenografts	[36,37]
Chromatin/hypoxia link	BAP1–H2A–SLC7A11	Pro-ferroptosis	BAP1 loss reduces erastin sensitivity under hypoxia; modifies RT response	NPC cell lines; xenografts	[38]
Protein methylation	PRMT4–Nrf2/GPX4 pathway	Anti-ferroptosis	Enhances cisplatin resistance; PRMT4 knockdown restores ferroptosis	NPC lines; xenografts	[39]
RNA acetylation	NAT10–SLC7A11 (ac4C modification)	Anti-ferroptosis	Stabilizes SLC7A11, confers resistance to sorafenib and platinum	In vitro NPC; xenograft	[40]
Viral oncogenesis	EBV-driven GPX4 upregulation; p62–Keap1–Nrf2	Anti-ferroptosis	Enhances antioxidant defense; drives progression and chemoresistance	Clinical samples; xenografts	[25]
Microenvironmental signals	CAF–FGF5–FGFR2–Nrf2–HO-1; Platelet-EV ITGB3	Anti-ferroptosis	Inhibits cisplatin-induced ferroptosis; promotes metastasis	NPC co-culture; in vivo	[41,42]

Abbreviations: ACSL4, acyl-CoA synthetase long-chain family member 4; CAF, cancer-associated fibroblasts; CP, ceruloplasmin; EBV, Epstein–Barr virus; EV, extracellular vesicle; HO-1, heme oxygenase-1; IGF2BP2, insulin-like growth factor 2 mRNA-binding protein 2; NPC, nasopharyngeal carcinoma; PUFA, polyunsaturated fatty acids; TXNIP, thioredoxin-interacting protein; ZnPP, zinc protoporphyrin IX. ↓ and ↑ indicated decreased and increased, respectively.

**Table 2:** Pharmacological and natural agents that induce ferroptosis in NPC.

Agent/Class	Primary Target or Pathway	NPC Model/Setting	Key Outcome	Combination Read-Through	Reference(s)
Erastin	System xCT inhibitor → ↓ cystine/GSH	CDDP-resistant HK1, C666-1; xenografts	Restored cisplatin cytotoxicity; ↓ tumor growth	Pairs with cisplatin for re-sensitization	[36,48]
RSL3	GPX4 inhibitor induced ↑ lipid peroxides	CNE2; H&N models	Inhibits survival; reverses resistance	Synergy with RT or COX-2/EGFR inhibitors	[55,69]
Sulfasalazine	xCT blockade (FDA-approved scaffold)	NPC cell lines	Depletes cystine/GSH; induces ferroptosis	Potential radiosensitizer; PD-1 synergy	[70,71]
Sorafenib	xCT/GSH depletion + kinase inhibition	NPC preclinical models	Sensitizes to RT and CDDP	Enhances RT/chemotherapy efficacy	[40,72]
Disulfiram/Cu	ROS/MAPK & ferroptosis via GSH depletion	NPC cell lines & CAFs	Antitumor activity against NPC cells + CAFs	Repurposed; combinable with CDDP	[48]
ZnPP (HO-1 inhibitor)	Blocks anti-ferroptotic HO-1	Cisplatin-resistant NPC; xenografts	Restores cisplatin sensitivity; ↓ tumor burden	Combines effectively with cisplatin	[36]
Solasodine	↑ Fe^2+^/ROS/MDA, ↓ GPX4/SLC40A1	NPC cell lines	Induces ferroptosis, suppresses growth	Potential complement with RT/chemotherapy	[28]
Berberine	Inhibits xCT–GSH–GPX4 axis	NPC in vitro/in vivo	↓ GPX4, ↓ SLC7A11; anti-metastatic	Radiosensitization reported	[26]
Cucurbitacin B	↑ iron, ↓ GPX4/GSH	NPC xenografts	Induces ferroptosis; enhances cisplatin efficacy	Works synergistically with CDDP	[27]
Celastrol + Curcumin	↑ ACSL4, ↓ GPX4/SLC7A11	NPC xenografts	Synergistic tumor suppression; low toxicity	Potentiates chemotherapy efficacy	[29]
Luteolin	SOX4/GDF15 suppression induced ↓ GPX4	NPC cell lines	Increases lipid ROS; enhances ferroptosis	Candidate radiosensitizer	[73]
Isoquercitrin	AMPK/NF-κB signaling	NPC cells; xenografts	Induces oxidative stress; tumor suppression	Dietary adjunct potential	[74]
Allicin	↓ GPX4/GSH, ↑ lipid ROS	HONE-1, HNE1 cells	Suppresses proliferation via ferroptosis	Candidate dietary sensitizer	[75]
Lupeol	Inhibits GPX4; ↑ ROS	NPC cell lines	Induces ferroptosis + apoptosis	Promising natural sensitizer	[76]
Itraconazole	↑ lysosomal iron; ferroptosis induction	NPC cells	Reduces stemness; ↑ lipid peroxidation	Maintenance therapy potential	[50]
Cephalosporins	HMOX1 activation → ferroptosis	NPC cells	Selective NPC killing	Drug repurposing candidate	[51]
PRMT4 inhibitors	Target PRMT4–Nrf2/GPX4 pathway	Cisplatin-resistant NPC	Restore ferroptosis; ↑ cisplatin efficacy	Synergy with CDDP	[39]
NAT10 inhibitors	Block ac4C–SLC7A11 stabilization	NPC models	Promote ferroptosis; overcome sorafenib resistance	Enhance platinum/sorafenib therapy	[40]
Nanoplatforms (Bi_2_Se_3_ hydrogels, SPIO-Erastin NPs)	ROS release, iron delivery, GPX4 suppression	NPC xenografts	Spatially controlled ferroptosis + apoptosis; tissue repair	Integrate with RT or PD-1 blockade	[30,77]
FSP1 inhibitors	Block ubiquinol regeneration	NPC models (preclinical)	Promote ferroptosis independent of GPX4	Candidate for combination with RT/ICI	[20,78]

Abbreviations: CDDP, cisplatin; ICI, immune checkpoint inhibitor; MDA, malondialdehyde; NPs, nanotherapeutic platforms; ROS, reactive oxygen species; RT, radiotherapy; SPIO, superparamagnetic iron oxide. ↓ and ↑ indicated decreased and increased, respectively.

**Table 3:** Biomarkers for stratifying patients for ferroptosis-targeted therapy in NPC.

**Biomarker**	**Assay/Sample Type**	**Directionality & Interpretation**	**Clinical Use Case**	**Reference(s)**
SLC7A11 (xCT)	IHC/RNA-seq	High → anti-ferroptotic; resistance risk	Predict radio/chemoresistance; exclude monotherapy inducers	[32,33]
GPX4	IHC/RNA-seq	High → resistance; Low → susceptibility	Select patients for GPX4-targeted strategies (e.g., RSL3)	[39,63]
ACSL4	IHC/RNA-seq	High → pro-ferroptotic lipidome	Radiosensitization candidate; marker of ferroptotic vulnerability	[22]
TFRC, FTH1, SLC40A1	RNA-seq/WGCNA	Iron metabolism-related FRGs; correlate with prognosis	Risk stratification based on iron metabolism	[37,81]
TXNIP	IHC in tumor biopsies	Low in resistant tumors; restoration ↑ ferroptosis	Predict radiosensitization potential	[31]
HO-1 (HMOX1)	IHC/qRT-PCR	High → cisplatin resistance via ferroptosis blockade	Add HO-1 inhibitor (ZnPP) with cisplatin	[36]
METTL3 (m^6^A writer)	RNA-seq/protein assay	Stabilizes SLC7A11 → suppresses ferroptosis	Predict RT resistance; candidate for epigenetic targeting	[33]
IGF2BP2 (m^6^A reader)	RNA-seq/IHC	Stabilizes CP mRNA; anti-ferroptotic	Prognostic biomarker; iron metabolism regulator	[23]
HOXA9–UBR5–SIRT6 axis	Protein assay/functional validation	Anti-ferroptotic; promotes progression	Therapy resistance marker; target for intervention	[24]
NAT10 (RNA acetyltransferase)	RNA/protein assays	Stabilizes SLC7A11 via ac4C → anti-ferroptotic	Marker for sorafenib/platinum resistance	[40]
EBV DNA (plasma)	qPCR	High baseline or slow decline → poor prognosis	Widely validated clinical biomarker; may reflect ferroptotic vulnerability	[4]
Immune infiltration signatures (M1 macrophages, neutrophils)	Bulk RNA-seq/IHC	High FRG activity linked to pro-inflammatory immune context	Predict response to ferroptosis-immunotherapy combinations	[37,59,82]
Dynamic PD markers (↓ GSH, ↑ MDA, ↑ Fe^2+^)	Tumor tissue assays/liquid biopsy	Real-time readout of ferroptosis induction	On-treatment monitoring and adaptive decision-making	[83,84]
FRG hub genes (TBK1, KIF20A, SLC16A1, QSOX1)	Multi-omics (WGCNA, RNA-seq)	Diagnostic/prognostic; link to immune infiltration	Baseline stratification for ferroptosis-targeted therapy	[37]

Abbreviations: FRG, ferroptosis-related gene; IHC, immunohistochemistry; PD, pharmacodynamic; qPCR, quantitative polymerase chain reaction; WGCNA, weighted gene co-expression network analysis. ↓ and ↑ indicated decreased and increased, respectively.

**Table 4:** Trial-ready combination concepts for ferroptosis in NPC (design scaffolds).

Setting	Concept Combo	Primary Endpoints	Stratifiers & On-Treatment Markers	Rationale
Locally advanced NPC (good induction responders)	CCRT ± low-dose erastin/sulfasalazine or HO-1 inhibitor during weeks 1–4	3-y DFS; ORN/late toxicity	Baseline: SLC7A11, GPX4; On-treatment: EBV DNA kinetics, GSH ↓/MDA ↑/Fe^2+^ ↑	Radiosensitization by tipping redox balance toward ferroptosis while minimizing normal tissue toxicity [4,13]
Recurrent/metastatic NPC (first-line)	Platinum doublet + PD-1 ± ferroptosis enabler (e.g., ACSL4 upmodulator, FSP1 inhibitor)	PFS, ORR; immune-related AEs	PD-L1, EBV DNA load, ferroptosis-related gene (FRG) signature	PD-1 combinations are standardizing; ferroptosis enhances immunogenic cell death and T cell priming [20,88]
Cisplatin-resistant disease	Cisplatin ± ZnPP (HO-1 inhibitor) or NAT10/PRMT4 inhibitor	ORR, PFS; nephrotoxicity monitoring	HO-1 expression, NAT10/PRMT4 activity, EBV DNA kinetics	Restores ferroptosis by blocking anti-ferroptosis defenses; overcomes platinum resistance [36,39,40]
Post-operative/bed therapy or salvage RT site	NIR-triggered Bi_2_Se_3_ nanosheet–alginate hydrogel	Local control; wound complications	Local MDA/4-HNE staining; MRI radiomics	Spatially selective ferroptosis with concurrent anti-inflammatory/tissue healing benefits [30]
Maintenance/secondary prevention	Itraconazole or disulfiram/Cu with low-dose RT/chemo	Time to recurrence; QoL	Iron metabolism genes (TFRC, FTH1), GSH levels	Repurposed agents induce ferroptosis and reduce stemness, potentially delaying relapse [48,50]

Abbreviations: AEs, adverse events; CCRT, concurrent chemoradiotherapy; DFS, disease-free survival; 4-HNE, 4-hydroxynonenal; MDA, malondialdehyde; QoL, quality of life; ORN, osteoradionecrosis; ORR, overall response rate; PFS, progression-free survival. ↓ and ↑ indicated decreased and increased, respectively.

## Data Availability

No new data were created or analyzed in this study. Data sharing is not applicable to this article.

## Abbreviations

ac4C, *N*4-acetylcytidine; ACSL4, acyl-CoA synthetase long-chain family member 4; ALOX, lipoxygenase; BH_4_, tetrahydrobiopterin; CAF, cancer-associated fibroblast; CAV1, caveolin-1; COX, cyclooxygenase; DAMPs, damage-associated molecular patterns; DHODH, dihydroorotate dehydrogenase; EBV, Epstein–Barr virus; ESCRT-III, endosomal sorting complexes required for transport-III; EV, extracellular vesicle; FSP1, ferroptosis suppressor protein-1; FTH1, ferritin heavy chain; GCH1, GTP cyclohydrolase-1; GPX4, glutathione peroxidase 4; GSH, glutathione; GSTM3, glutathione *S*-transferase mu 3; HAT1, histone acetyltransferase 1; 4-HNE, 4-hydroxynonenal; HO-1, heme oxygenase-1 (HMOX1); IGF2BP2, insulin-like growth factor 2 mRNA binding protein 2; LPCAT3, lysophosphatidylcholine acyltransferase 3; m^6^A, *N*6-methyladenosine; MDA, malondialdehyde; NAD(P)H/NAD(P)^+^, reduced/oxidized nicotinamide adenine dinucleotide (phosphate); NAT10, *N*-acetyltransferase 10; NCOA4, nuclear receptor coactivator 4; NPC, nasopharyngeal carcinoma; NRF2, nuclear factor erythroid-2-related factor 2; OTUB1, otubain-1; PCK2, phosphoenolpyruvate carboxykinase 2; P5HA1, prolyl 4-hydroxylase subunit alpha 1; POR, cytochrome P450 oxidoreductase; PRMT4, protein arginine methyltransferase 4; PUFA, polyunsaturated fatty acid; ROS, reactive oxygen species; RT, radiotherapy; SLC7A11, solute carrier family 7 member 11 (light chain of system x_c_^−^); system x_c_^−^, cystine/glutamate antiporter (xCT); SOD2, superoxide dismutase 2; TFRC, transferrin receptor; TME, tumor microenvironment; TXNIP, thioredoxin-interacting protein; ZnPP, zinc protoporphyrin.
